# Fluoroquinolone Prescribing in Primary Care: Patient Safety, Antimicrobial Stewardship and Forensic Implications Under the Italian Framework—A Narrative Review

**DOI:** 10.3390/pharmacy14070153

**Published:** 2026-10-19

**Authors:** Giulia Ciancarella, Pasquale Ricci, Serafino Ricci, Noman Al Ani, Pietro Tasegian, Arnaldo Miglino, Lidia Ricci

**Affiliations:** 1 Department of Anatomical, Histological, Forensic and Orthopaedic Sciences, University “Sapienza” of Rome, Piazzale Aldo Moro 1, 00185 Rome, Italy; 2 Primary Care Department, Azienda Sanitaria ASL Roma 2, 00159 Roma, Italy; 3 Department of Life Science, Health and Health Profession, Link Campus University, Via del Casale di San Pio V, 44, 00165 Rome, Italy; 4 Primary Care Department, Azienda Sanitaria USL Umbria 1, 06012 Città di Castello, Italy; 5 Independent Researcher, 00100 Rome, Italy; 6 Ministry of Labour and Social Policies, Via Vittorio Veneto 56, 00187 Rome, Italy

**Keywords:** fluoroquinolones, patient safety, antimicrobial stewardship, primary care, pharmacists, informed consent, off-label use, professional liability, Italy

## Abstract

Fluoroquinolones remain widely prescribed in primary care although regulators have restricted their use because of rare but serious, potentially disabling and irreversible adverse reactions. This narrative review, based on a PubMed search (2008–2026) and regulatory and Italian legal sources, examines fluoroquinolone safety, prescribing patterns and forensic implications from a general-practice and legal-medicine perspective. The evidence behind regulatory warnings is uneven: tendon and aortic events have the strongest observational support, other associations remain uncertain, and absolute risks are low. The US Food and Drug Administration and the European Medicines Agency advise against fluoroquinolones for mild or self-limiting infections when alternatives exist, yet US, European and Italian data show persistent use, particularly for uncomplicated cystitis. The forensic analysis is limited to the Italian framework (Laws no. 24/2017 and no. 219/2017) and cannot be generalised to other jurisdictions: prescribing that departs from the product information or from guidelines is not in itself negligent, and liability depends on the individual case, clinical reasoning, information given and documentation. General practitioners and pharmacist-led stewardship, prescription review and patient counselling can support appropriate use. Fluoroquinolones should be reserved for, not withheld from, patients in whom they are clinically indicated. A proposed, non-validated checklist for prescribing and documentation is provided.

## 1. Introduction

Fluoroquinolones are one of the most widely prescribed antibiotic classes worldwide, reflecting their broad antimicrobial activity, favourable pharmacokinetic profile, and ease of administration [1]. Fluoroquinolones are classified in the ‘Watch’ group of the World Health Organization (WHO) AWaRe (Access, Watch, Reserve) classification [2]. This category includes broad-spectrum agents considered to be second-line treatments, with a higher inherent risk of inducing bacterial resistance. According to antimicrobial stewardship guidelines, their use should be restricted to specific infectious conditions and only when other options with a lower impact on resistance are not appropriate [2].

In ambulatory care they have long been among the most frequently ordered antibiotics, mainly for urinary and respiratory tract infections. Much of this use rests on a long-standing perception that the class is safe and reliable [3].

That perception has changed. Fluoroquinolones are now linked to a distinctive group of serious, potentially disabling, and sometimes irreversible reactions. In response, the US Food and Drug Administration (FDA) issued its first boxed warning in 2008, expanded it in 2016 and required further labelling warnings in 2018, while the European Medicines Agency (EMA) and national regulators introduced parallel restrictions between 2018 and 2020 [4,5,6,7]. The shared message is clear: fluoroquinolones should be avoided for uncomplicated urinary tract infection (UTI), acute sinusitis, and acute exacerbation of chronic bronchitis when alternatives exist, because for these mild or self-limiting conditions the risks generally outweigh the benefits [7,8].

Translation into practice has been inconsistent. Outpatient prescribing has declined following regulatory warnings, but reductions have varied over time and between health systems, and prescribing has persisted for some of the indications targeted by regulators [9,10]. This gap is greatest in primary care, where fluoroquinolones are most often started. In Italy, in 2024, the share of fluoroquinolones in total antibiotic consumption stood at 9.3%. Although a steady downward trend has been observed in recent years, the Italian figure remains substantially higher than the European average [3]. The gap is not only a stewardship problem; it also defines an area of potential forensic exposure that has received little structured attention. When a medicine carries prominent regulatory warnings, restricted indications and identifiable high-risk groups, prescribing it outside these limits raises questions regarding the appropriateness of the indication, the adequacy of risk information and consent, the documentation of the risk–benefit balance, and the duty to provide advice and to discontinue the medicine promptly [11,12]. Where the use departs from the conditions set out in the summary of product characteristics (SmPC), the prescription may fall within the off-label regime, which in Italy requires that the patient be informed and that written consent be obtained, according to the Code of Medical Ethics [13,14]. Recent safety reviews [15] have focused primarily on epidemiology and pharmacovigilance but have not examined the implications for professional liability. To our knowledge, no review has integrated these perspectives for primary care.

Written from the combined perspective of general practice and legal medicine, this review aims to bridge that gap. Its objectives are: (1) to synthesise current evidence on fluoroquinolone harms and the FDA and EMA framework, distinguishing established regulatory warnings from associations that rest mainly on observational evidence; (2) to examine the discrepancy between guidance and real-world prescribing in primary care, keeping Italian data separate from those of other health systems; (3) to clarify how regulatory provisions, clinical guidelines and legal norms bear on the assessment of professional liability; and (4) to translate these into a practical approach to prescribing, risk communication, and documentation, including the contribution of pharmacists, summarised in a proposed, non-validated prescriber-oriented checklist. Because the rules on professional liability, informed consent and off-label use are specific to each jurisdiction, the forensic analysis is confined to the Italian legal framework and should not be generalised to other health systems without adaptation; the safety and prescribing evidence is drawn from international sources, and its geographic origin is stated throughout.

## 2. Methods

This is a narrative review. Its aim was to bring together, in a single clinical and forensic overview, the evidence on fluoroquinolone safety, the related regulatory decisions, prescribing habits in primary care, and the legal implications for prescribers. It is not a systematic review, and no statistical pooling was performed; its reporting was guided by the Scale for the Assessment of Narrative Review Articles (SANRA) [16]. We searched PubMed for articles published between 2008, the year of the first FDA boxed warning on fluoroquinolones, and 2026. The search combined the terms “fluoroquinolones” and “quinolones” with the names of individual drugs (ciprofloxacin, levofloxacin, and moxifloxacin) and with safety- and practice-related terms including “adverse effects”, “tendon rupture”, “peripheral neuropathy”, “aortic aneurysm”, “boxed warning”, “prescribing”, “primary care”, and “informed consent”. The full search strategy was: (“fluoroquinolones” OR “quinolones” OR “ciprofloxacin” OR “levofloxacin” OR “moxifloxacin”) AND (“adverse effects” OR “tendon rupture” OR “peripheral neuropathy” OR “aortic aneurysm” OR “boxed warning” OR “prescribing” OR “primary care” OR “informed consent”), with the publication date limited to 2008–2026. The last search was performed on 15 August 2026 and retrieved 57,886 records.

Because the search strategy was deliberately broad, the 57,886 records retrieved from PubMed were not screened exhaustively, as would be required for a systematic review. Instead, a purposive, relevance-based selection process was adopted. Records were examined in order of PubMed’s “Best Match” ranking. Titles and abstracts were assessed by one author (G.C.) for relevance to at least one of the four review questions: safety, regulation, prescribing in outpatient or primary care, and forensic implications. Screening was not independently duplicated by a second reviewer, and inter-rater agreement was not assessed. The selection of sources for inclusion was discussed among the authors, and any disagreements were resolved by consensus. When several sources addressed the same question, priority was given to systematic reviews and meta-analyses, large population-based studies, and the most recent relevant publications. As part of the review, P.T. conducted additional targeted PubMed searches on 3 October 2026 concerning the role of pharmacists in antimicrobial management, evidence on individual adverse reactions, and prescribing data from Italy and other European countries. Prescribing studies were classified by country to allow Italian, other European, and US evidence to be reported separately. The synthesis draws on 77 sources: 54 journal articles, 13 regulatory or institutional documents and clinical guidelines, 8 legal sources and two textbooks. No formal risk-of-bias assessment was performed.

We also consulted the official websites of the main regulatory agencies—the FDA, the EMA, the UK Medicines and Healthcare products Regulatory Agency (MHRA), and the Italian Medicines Agency (AIFA)—for the original safety communications, product information, and warnings, and we checked the reference lists of the articles we found for further relevant papers. Italian statutes, ministerial decrees and the Code of Medical Ethics were consulted in their official texts; case law was retrieved from the DeJure database (Giuffrè Francis Lefebvre) and selected by the authors with legal and forensic expertise P.R., S.R., A.M. and L.R.

We included studies, reviews, guidelines, and regulatory documents on fluoroquinolone safety, prescribing, or forensic aspects that were relevant to everyday outpatient and primary care practice, published in English or Italian. We excluded articles limited to eye or skin formulations and those concerning only highly specialised hospital settings.

The authors discussed the selected material together, each bringing their own perspective. The findings were grouped by theme and are presented below, ending with a proposed checklist. As the review uses only published, publicly available material, ethical approval was not required.

The authors used OpenEvidence (2026), an artificial intelligence (AI)-based medical information platform, as an adjunct to the PubMed search to identify potentially relevant literature, and to support the drafting and language editing of parts of the manuscript. Every source suggested by the tool was retrieved and checked by the authors against its PubMed record and full text before inclusion, and no source was cited based on an AI-generated summary alone. The tool was not used to decide which studies to include, to interpret the evidence, or to formulate the forensic analysis and the conclusions. During revision, the authors also used Claude (Anthropic, San Francisco, CA, USA; model Claude Opus 5.5; 3–4 October 2026), a generative AI model, to conduct the additional targeted PubMed searches described above, to cross-check the reference list against PubMed records, and to assist in the preparation of graphical figures. The sources identified through these searches were independently verified against their corresponding PubMed records and abstracts. The authors reviewed and edited all AI-assisted content and visual materials and take full responsibility for the content and accuracy of this publication.

## 3. Fluoroquinolone Safety and the Regulatory Response

### 3.1. The Main Safety Concerns

Fluoroquinolones are commonly associated with mild and reversible adverse effects, such as nausea, diarrhoea, and headache. However, accumulating evidence has established that this drug class can also cause serious adverse reactions that may be disabling and persist long after treatment discontinuation [17]. The current boxed warning highlights tendinitis and tendon rupture, peripheral neuropathy, central nervous system effects, and worsening of muscle weakness in myasthenia gravis [6,18]. These reactions are not all established to the same degree. Table 1 presents the principal ones in two groups and keeps apart two aspects that are often conflated: the regulatory status of each reaction and the type and strength of the evidence behind it.

Tendon injury is the best-known reaction. It most often involves the Achilles tendon and can appear in the first days of treatment or weeks after the drug is stopped; the risk is higher in older patients, those on corticosteroids, and those with kidney problems [7,19]. In a meta-analysis of 15 observational studies, fluoroquinolones were associated with Achilles tendon rupture (odds ratio (OR) 2.52, 95% confidence interval (CI) 1.81–3.52) and with any tendon disorder (OR 1.98, 95% CI 1.62–2.43) [20].

The class has also been linked to peripheral nerve damage, which can be irreversible, and to psychiatric and central nervous system effects [21]. These reactions appear in the warnings on both sides of the Atlantic, but the supporting evidence comes largely from spontaneous reports and observational studies. In a UK primary-care nested case–control study, current oral fluoroquinolone exposure was associated with peripheral neuropathy (adjusted incidence rate ratio 1.47, 95% CI 1.13–1.92), with an absolute risk of 2.4 per 10,000 patients per year of current use [22]. In a German claims-based cohort, relative risks for neurological and neuropsychiatric outcomes compared with other antibiotics ranged from 1.04 to 1.10 [23], and an umbrella review found no systematic review with meta-analysis on neuropsychiatric toxicity or long-term disability [24]. Other recognised problems include QT prolongation and arrhythmia and changes in blood sugar; older patients and those with diabetes or heart disease appear more vulnerable [25,26].

A further group of associations emerged from pharmacoepidemiological studies and was then incorporated into regulatory warnings: aortic aneurysm and dissection and, later, heart valve regurgitation [27,28,29]. For aortic events, an umbrella review rated the association as highly suggestive and supported by evidence of causality [24], whereas a later propensity-matched cohort pointed to residual confounding and surveillance bias (Section 7.2) [30]. For valve regurgitation, the warning followed a single pharmacoepidemiological study whose authors called for confirmation [31]. For retinal detachment the evidence is conflicting: a meta-analysis did not confirm the association, and the umbrella review rated the evidence of causality as weak [24,32].

**Table 1:** Principal serious adverse reactions associated with systemic fluoroquinolones: regulatory status and strength of the underlying evidence.

Adverse Reaction	Regulatory Status	Evidence Base and Remarks
***A. Long-established class warnings***		
Tendinitis and tendon rupture	FDA: boxed warning (2008). EU: warning in the product information; one of the reasons for the 2018–2019 restrictions [6,7]	Consistent observational evidence. Meta-analysis of 15 studies: Achilles tendon rupture OR 2.52 (95% CI 1.81–3.52); credibility rated ‘highly suggestive’ in an umbrella review. Higher risk with older age, corticosteroids, renal impairment and organ transplantation [7,20,24]
Exacerbation of myasthenia gravis	FDA: boxed warning. EU: warning in the product information [6]	Mainly post-marketing reports; no pooled estimate identified
Peripheral neuropathy	FDA: labelling strengthened in 2013; included in the boxed warning in 2016. EU: warning in the product information [6,7,9]	Single large observational study (UK primary care, nested case–control): adjusted incidence rate ratio 1.47 (95% CI 1.13–1.92); absolute risk 2.4 per 10,000 patients per year of current use. May be irreversible [22]
Central nervous system and psychiatric effects	FDA: boxed warning (2016); labelling for mental-health effects strengthened in 2018. EU: warning in the product information [6,7,33]	Spontaneous reports and observational cohorts; relative risks versus other antibiotics 1.04–1.10 in a German claims cohort; no meta-analysis available [21,23,24]
Dysglycaemia, including severe hypoglycaemia	FDA: labelling strengthened in 2018. EU: warning in the product information [33]	Population-based cohort of patients with diabetes (Taiwan): higher risk of severe hypo- and hyperglycaemia than with macrolides, greatest for moxifloxacin [25,26]
QT prolongation and arrhythmia	FDA and EU: warning in the product information [25]	Review of descriptive and aetiological studies: risk differs between agents and is increased by predisposing factors such as heart disease and interacting medicines [25]
***B. Warnings and associations that rest mainly on spontaneous reports or on recent observational studies***		
Disabling, long-lasting and potentially irreversible reactions involving several body systems	FDA: boxed warning (2016). EU: EMA review concluded in 2018, binding European Commission decision in 2019, reminder to prescribers in 2023 [6,7,34]	Mainly spontaneous reports assessed by the regulators, who describe the reactions as very rare; frequency not reliably quantified; no meta-analysis available [17,24,35]
Aortic aneurysm and dissection	FDA: warning added in December 2018. EU: warning communicated to prescribers in October 2018 [27,28]	Observational studies only. Meta-analysis: OR 1.47 (95% CI 1.13–1.89) for aneurysm or dissection, about 7246 patients treated for one additional event; credibility ‘highly suggestive’ in the umbrella review. In a later propensity-matched cohort the association was present in pneumonia but not in UTI and was attenuated when baseline imaging was required; absolute rates below 0.1% [24,30,36]
Heart valve regurgitation	EU: warning communicated to prescribers in October 2020. Not addressed in the FDA communications reviewed here [29]	Single disproportionality analysis and nested case–control study (adjusted rate ratio 2.40 versus amoxicillin, 95% CI 1.82–3.16); the authors called for confirmatory studies [31]
Retinal detachment	Not the subject of a specific warning in the regulatory communications reviewed here	Conflicting observational studies; meta-analysis did not confirm an association; evidence of causality rated weak [24,32]

FDA, US Food and Drug Administration; EU, European Union; EMA, European Medicines Agency; OR, odds ratio; CI, confidence interval; UTI, urinary tract infection. The grouping into A and B is the authors’ qualitative summary, not a formal grading of evidence. FDA labelling has no legal effect in the European Union and is reported here as a source of safety information. Estimates come from observational studies and should not be read as individual risks.

### 3.2. The Regulatory Response

In the United States, the FDA issued its first boxed warning in July 2008 for tendinitis and tendon rupture, added a warning about irreversible peripheral neuropathy in 2013, and in 2016 recommended that fluoroquinolones not be used as first-line treatment for uncomplicated UTI, acute sinusitis, and acute exacerbation of chronic bronchitis when other options exist [4,6,9,19]. Further warnings in 2018 covered low blood sugar, mental health effects, and aortic aneurysm and dissection [17,27,33]; these changes were made in the warnings sections of the labelling, not in the boxed warning.

**Figure 1:**
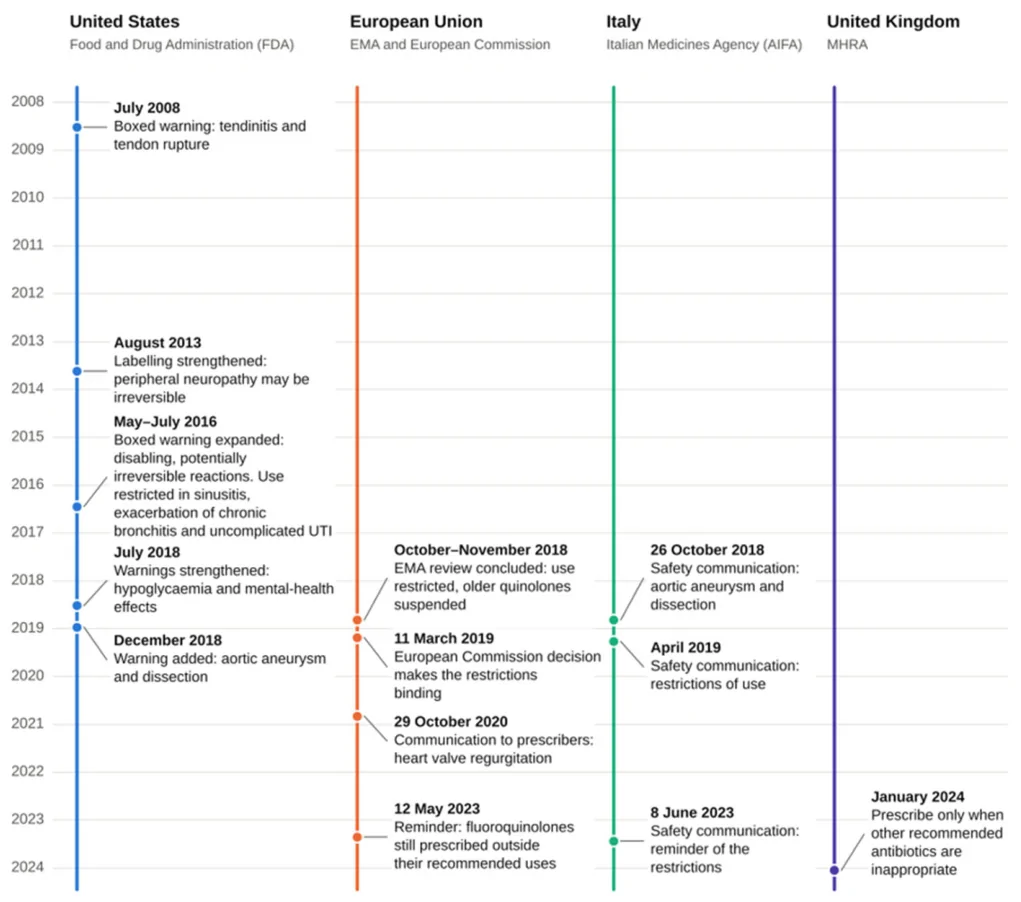
Regulatory actions on systemic fluoroquinolones in the United States, the European Union, Italy and the United Kingdom, 2008–2024. The vertical axis is proportional to time. FDA, US Food and Drug Administration; EMA, European Medicines Agency; AIFA, Italian Medicines Agency; MHRA, Medicines and Healthcare products Regulatory Agency; UTI, urinary tract infection. Sources: [4,6,7,9,19,27,28,29,33,34,35,37,39,40].

Europe followed a similar path. Following a formal review concluded in 2018, the EMA found that fluoroquinolones may cause long-lasting, disabling, and potentially irreversible adverse reactions and recommended restricting their use, particularly for conditions in which the expected benefit is limited or for which safer therapeutic alternatives are available [7]. Under the resulting European Commission decision of March 2019, fluoroquinolones should not be used for infections that are not severe or might resolve without treatment, for non-bacterial infections, or to prevent traveller’s diarrhoea or recurrent lower UTI; for mild or moderate bacterial infections they should be used only when other commonly recommended antibacterials cannot be used, and with particular caution in older patients, in those with kidney disease or an organ transplant, and in those taking corticosteroids [7]. These recommendations were communicated to prescribers through updated product information and direct healthcare-professional communications, including communications issued by AIFA in Italy [37]; separate communications concerned aortic aneurysm and dissection (October 2018) and heart valve regurgitation (October 2020) [28,29]. In 2023 the EMA and AIFA reminded prescribers of the restrictions after an EMA-funded study in six European countries which, according to the agency, suggested that fluoroquinolones may still be prescribed outside their recommended uses [34,35]; the published analysis found no relevant effect of the 2018–2019 regulatory action on prescribing in primary care [10]. In clinical practice, this risk–benefit assessment is particularly relevant for conditions such as uncomplicated UTI, acute sinusitis, and acute exacerbation of chronic bronchitis, where fluoroquinolones should generally be avoided when effective and safer alternatives are available. For uncomplicated UTI, however, the possibility of spontaneous symptom resolution should not be equated with an absence of risk, as non-antibiotic management has been associated with a small but clinically relevant risk of progression to pyelonephritis [38]. Furthermore, in January 2024, the MHRA further restricted the use of systemic fluoroquinolones, recommending that they be prescribed only when other commonly recommended antibiotics are inappropriate and following careful consideration of the risks and benefits [39,40]. The sequence of regulatory actions is summarised in Figure 1.

The core message from the agencies is the same: for common, self-limiting infections the serious risks generally outweigh the benefits, and fluoroquinolones should be reserved for cases with no suitable alternative. These are regulatory provisions that define the authorised conditions of use; they are not clinical guidelines, and their legal significance in Italy is examined in Section 5.

## 4. The Gap Between Recommendations and Everyday Prescribing

### 4.1. Warnings Have Reduced Use, but Only in Part—Focus on Italy

In 2024, fluoroquinolone consumption in Italy amounted to 1.4 defined daily doses (DDD) per 1000 inhabitants per day, accounting for 9.3% of total antibiotic consumption and ranking fourth among antibiotic classes. Fluoroquinolone consumption decreased by 9.3% compared with 2023 and by 43.2% compared with 2018 [3].

Approximately 90% of total publicly funded antibiotic consumption occurred through contracted outpatient care. This identifies general practice as the main setting for antibiotic prescribing, including fluoroquinolones, and a key target for antimicrobial stewardship [3]. Fluoroquinolone use varies considerably across Italian regions. Consumption is highest in Southern Italy (1.9 DDD per 1000 inhabitants per day), despite the largest year-on-year decline (11.6%), is more than twice that observed in Northern Italy (0.9 DDD per 1000 inhabitants per day). Central Italy, with 1.5 DDD per 1000 inhabitants per day, is closer to the national average. Regarding population subgroups:

- Older adults (aged ≥ 75): This is a high-risk group with a prevalence of use of 12.8% and consumption of 3.3 DDD per 1000 inhabitants per day. Despite an 11% reduction compared with 2021, usage remains high, particularly in the South (prevalence 19.5%).
- Women (aged 20–59): They show a prevalence of use of 4.9%, with levels in the South twice those in the North.
- Private purchases: Ciprofloxacin and levofloxacin rank among the top 10 active substances by per capita expenditure in private Class A purchases, indicating substantial use even outside the National Health Service (SSN) reimbursement scheme [3].

The reduction, however, is incomplete and fluoroquinolones remain widely prescribed antibiotics, although their use is not always consistent with current recommendations regarding appropriate indications and antibiotic choice. In June 2023 AIFA reminded prescribers that these medicines were still being prescribed outside their recommended uses [35]. Italian data also show the ecological cost of community use: in a case–control study from the Autonomous Province of Bolzano, fluoroquinolone use in primary care was associated with later isolation of fluoroquinolone-resistant *Escherichia coli* during a hospital stay; the risk was highest in the first year after exposure (OR 2.67, 95% CI 1.92–3.70) and was still detectable in the second year [41].

### 4.2. Other European Health Systems

Evidence from other European countries points in the same direction but is heterogeneous. An EMA-funded study of electronic health records from six countries (2016–2021) found that changes in fluoroquinolone prescribing in primary care were inconsistent and not clearly related in time to the 2018–2019 regulatory action [10,34]. In the Netherlands and the United Kingdom the same measures were followed by a moderate decline in incident use [42], and in England primary-care dispensing fell by 4.2% per year between 2019 and 2023 [40]. In French general practice, fluoroquinolone prescriptions fell by 59% between 2014 and 2023, yet fewer than 25% of prescriptions complied with the EMA 2018–2019 restrictions [43]. An analysis of the EudraVigilance database found no significant change in reported musculoskeletal and nervous-system reactions after the EMA warning [5].

### 4.3. Uncomplicated UTI Is the Main Problem Area: US and Italian Data

The largest gap concerns uncomplicated UTI in women, where guidelines recommend nitrofurantoin, trimethoprim-sulfamethoxazole, or fosfomycin and specifically discourage fluoroquinolones [44,45]. Most of the quantitative evidence on guideline concordance comes from the United States. In a US analysis covering 2015 to 2019, fluoroquinolones were still the single most prescribed class for uncomplicated UTI (36.4% of visits). Concordance with the Infectious Diseases Society of America guidelines improved over the study period (from 48.2% to 64.6%), but was only 58.4% overall [46]. Among commercially insured US patients, the share of fluoroquinolones in antibiotic-treated episodes of uncomplicated UTI fell from 41.6% in January 2015 to 19.2% at the end of 2018 [4]. These figures come from a health system with different organisation, reimbursement and reference guidelines, and they should not be read as estimates for Italy. Italian evidence at the level of the indication is more limited. In the multi-country REWIND (REal World INternational Database) study, a retrospective cohort study based on primary-care electronic medical records from Italy and Belgium, covering the period from 1 January 2016 to 31 December 2017, fosfomycin trometamol was the first-choice antibiotic for UTIs in women, whereas ciprofloxacin was prescribed to 24.6% of antibiotic-treated patients in Italy [47]. These data therefore reflect prescribing practices preceding the 2019 European regulatory restrictions on fluoroquinolone use. More recent indication-level data from Italian general practice were not identified, which is a limitation of the evidence base.

### 4.4. Why the Gap Persists, and the Role of General Practitioners

Much of the explanation lies in prescriber beliefs rather than a lack of alternatives. In a qualitative study of 18 primary care providers in two US family medicine clinics, many believed that fluoroquinolones work faster and better than first-line agents for uncomplicated UTI [48]; their broad spectrum and convenient dosing may also make them an easy choice in a busy practice. Whether the same beliefs operate among Italian general practitioners has not, to our knowledge, been studied. General practitioners are central to efforts to improve fluoroquinolone prescribing. In a large US Medicare study, prescribing among primary care physicians declined by 1.34 percentage points more than among non-primary-care physicians following the 2016 safety warning, although overall prescribing had risen after the 2013 warning [9]; ambulatory fluoroquinolone use in the United States fell by 26.7% between 2015 and 2019, with an immediate drop after the 2016 advisories [49]. These findings suggest that regulatory warnings can influence prescribing behaviour, but that their effect differs between warnings and between groups of prescribers, and the persistence of established prescribing patterns may remain an important barrier to sustained improvement.

## 5. The Forensic Dimension Under the Italian Framework

### 5.1. Why Fluoroquinolones Raise a Specific Liability Question

Clear regulatory warnings on one side and continued prescribing for mild infections on the other create a potential forensic exposure. When the product information warns of serious, sometimes irreversible harm and restricts use to situations in which other commonly recommended antibacterials are inappropriate [7,17], a prescriber who departs from those conditions without recording the reasons may find the choice harder to justify if the patient is harmed (this depends on the rules governing the taking of evidence in court, not on a rule of substantive law).

According to the prestigious De Jure database (published by Giuffrè, one of the most widely used in the Italian legal sector), there is only one case in which the issue concerning the use of fluoroquinolones was brought to the attention of the Italian judiciary. The case was settled by the Civil Court of Novara in its judgement No. 129 of 2 April 2026 [in De Jure, the database of Giuffré Editore in Milan]. The judge stated, amongst other things: ‘A first element worthy of censure [against a care home] … was the administration, prior to the operation, of antibiotic prophylaxis with ciprofloxacin, a drug belonging to the fluoroquinolone class. The Guidelines on perioperative antibiotic prophylaxis in adults state that, in the case of cardiac surgery (aorto-coronary bypass), it is necessary to administer a first-generation cephalosporin (cefazolin 2 g) or a second-generation cephalosporin (cefuroxime 2 g) [13]. Incorrect antibiotic prophylaxis during surgery, in fact, increases the risk of developing surgical site infections; it is therefore considered that the antibiotic prophylaxis administered did not comply with the Guidelines and consequently led to an increased likelihood of a surgical site infection occurring’. Partly as a result of this clinical error (which, in truth, was not the only one), the care home and the relevant Local Health Authority, which were brought before the court, were ordered to pay compensation for the damage resulting from the injuries and suffering endured by the patient prior to his death.

Empirical data on claims are scarce: in a five-year review of Irish national incident and claims data, four finalised claims concerned bilateral Achilles tendon rupture after fluoroquinolone exposure, all in patients aged over 60, and in 15 of the 20 reported incidents the reporter judged the prescription inappropriate [50].

To keep the analysis precise, four kinds of source must be distinguished. They are related but not interchangeable, and each has a different status under Italian law (Table 2).

### 5.2. Prescribing That Departs from the Product Information or from Guidelines

The first issue is appropriateness. For a prescriber in Italy the relevant regulatory source is the European product information, which since 2019 has allowed fluoroquinolones for uncomplicated cystitis and other mild or moderate infections only when other commonly recommended antibacterials cannot be used [7,37]; the FDA position is concordant but has no legal effect in Italy. Guidelines contribute to defining the standard of care against which conduct is assessed [53]; a doctor may deviate from this for valid reasons, which should be documented in the patient’s medical records, for the reasons relating to the standard of proof already highlighted: an undocumented deviation could prove more difficult to justify at a later date. Firstly, it should be noted that the subject under consideration is governed by Law No. 24 of 8 March 2017, which establishes ‘Provisions concerning the safety of care and of the person receiving care, as well as concerning the professional liability of healthcare professionals’ (the so-called Gelli–Bianco Law) [11,55]. Article 5, paragraph 1, of that Law stipulates that “Healthcare professionals, when providing healthcare services for preventive, diagnostic, therapeutic, palliative, rehabilitative and forensic medical purposes, shall comply, subject to the specific circumstances of each individual case, with the recommendations set out in the guidelines published pursuant to paragraph 3 and drawn up by public and private bodies and institutions, as well as by scientific societies and technical-scientific associations of the healthcare professions included in a specific register established and regulated by a decree of the Minister of Health, to be issued within ninety days of the date of entry into force of this Act, and to be updated every two years. In the absence of the aforementioned recommendations, healthcare professionals shall adhere to good clinical and care practices.” This is a general provision which applies to all areas where medical liability arises. The Civil Court of Cassation, in judgement no. 13510/22 of Section III, clarified: ‘With regard to medical liability for medical and surgical practice, the so-called “soft law” of the guidelines—whilst not having the force of statutory law—constitutes a set of parameters for determining medical fault, which contribute to the correct classification of the specific case under the legal framework governed by general provisions.’

It is now worth clarifying the legal significance that an erroneous, imprudent or negligent prescription of fluoroquinolones holds under the various branches of Italian law.

With regard to criminal liability, Article 6 introduced Article 590-sexies of the Criminal Code, the second paragraph of which provides: “Where the event occurred as a result of lack of skill (*imperizia*), punishability is excluded where the recommendations set out in the guidelines, as defined and published in accordance with the law, or, in the absence of such guidelines, good clinical and care practices, are followed, provided that the recommendations set out in the aforementioned guidelines are appropriate to the specific circumstances of the case” (authors’ translation).

Two key points emerge from the text of the regulation. First, the exemption from liability applies solely to lack of skill (*imperizia*), not carelessness (*negligenza*) or imprudence (*imprudenza*); according to the Joint Criminal Divisions of the Court of Cassation, it applies to non-gross lack of skill in carrying out recommendations that were appropriately chosen for the case [52]. Second, it presupposes that the recommendations were followed: a prescriber who has departed from them cannot rely on it, and the conduct is assessed under the ordinary criteria of fault [11,52].

According to the Italian Court of Cassation (Third Civil Division, decision no. 13510 of 29 April 2022) [53], guidelines do not have immediate legal force but set out parameters for determining medical negligence. These parameters do not have absolute value. By virtue of the provisions referring to ‘the specific circumstances of the individual case’, the legal provisions not only do not automatically hold the doctor liable for deviating from the guidelines but indeed require the doctor not to adhere to these parameters when it is necessary to adopt the clinical approach best suited to safeguarding the patient’s health. The aforementioned Joint Criminal Chambers, in their judgment No. 8770 of 21 December 2017, filed on 22 February 2018 [52], stated the following: “With regard to the liability of healthcare professionals, the recommendations contained in the guidelines defined and published pursuant to Article 5 of Law no. 24 of 8 March 2017—whilst representing the pre-established parameters to which the court must generally adhere when assessing compliance with the obligations of diligence, prudence and expertise—do not constitute genuinely binding precautionary rules capable of constituting, in the event of a culpable breach, a case of specific negligence, given the necessary flexibility required to adapt them to the specific case; it follows that, where such recommendations are not adequate in relation to the objective of providing the best possible care for the patient’s specific case, the healthcare professional has a duty to deviate from them” (authors’ translation). This appears to be consistent with medical science, according to which “there are patients, not diseases: therefore, each disease may take a distinct course in every individual” [54].

The case law of the Civil Division of the Court of Cassation has aligned with that of the Criminal Division. Indeed, according to the aforementioned judgement No. 13510/22 of the Third Civil Section of the Court of Cassation, the guidelines set out the parameters for determining medical negligence, but they do not have absolute authority. By virtue of the provisions referring to the ‘specific circumstances of the individual case’, the law not only does not automatically attribute liability to the doctor for deviating from the guidelines, but indeed requires the doctor not to adhere to such parameters when it is necessary to adopt the clinical approach best suited to safeguarding the patient’s health.

A prescription that does not comply with the guidelines or the product information does not, in itself, constitute negligence. Non-compliance is a clinical and therapeutic management category. Fault is a legal category arising from the outcome of a judgement aimed at establishing whether the medical act fell below the standard expected in the specific circumstances. Liability presupposes that the patient has suffered harm and that a causal link between the conduct and the harm has been established in accordance with the standard of proof applicable to the proceedings in question. The assessment of whether liability exists therefore depends on the patient’s characteristics, the clinical reasoning, the alternatives actually available (allergy, intolerance, interactions, local resistance, previous treatment failure, microbiological results), the information provided and the documentation. Conversely, a serious adverse reaction may occur following a wholly appropriate prescription, and its occurrence does not in itself indicate fault (Section 7.2).

Therefore, for example, the choice of a fluoroquinolone as first-line treatment for uncomplicated UTIs is a decision which, in our view, requires a well-structured clinical rationale, preferably supported by validated and authoritative medical references, particularly when the product information and guidelines advise otherwise and there is no documented reason to rule out alternatives: only in this way this choice can be properly assessed in any subsequent legal assessment of negligence and causation [53,56].

Medical practice is governed not only by criminal and civil law, but also by the Code of Ethics, which is primarily inspired by the Hippocratic principle of ‘beneficence’ towards the patient [57] in addition to addressing conduct strictly relating to the ethical nature of healthcare professionals’ actions, essentially reaffirms (through the formulation of several provisions) the need for clinically sound conduct, free from negligence and incompetence. The Code is issued by the National Federation of Orders of Physicians, Surgeons and Dentists (FNOMCeO) and is enforced by each Professional Council to which the individual doctor is registered, through disciplinary proceedings following which the Council itself may impose the following sanctions (provided for in Article 40 of Presidential Decree No. 221 of 5 April 1950):

- A warning, which consists of admonishing the offender not to repeat the offence committed;
- A reprimand, which is a declaration of censure for the offence committed;
- Suspension from practising the profession for a period of between one and six months;
- Striking off the register.

Given the broad scope of the Code and the sanctions provided for by law, a prescription of fluoroquinolones—however ill-advised it may be from the perspective of criminal or civil law—is unlikely to fall outside the scope of the code of professional conduct, thereby directly affecting the professional practice of the individual deemed liable to sanctions.

There is no reason to rule out, as a matter of principle, any liability of an administrative and financial nature (so-called ‘liability to the public purse’) where the inappropriate use of fluoroquinolones causes financial loss to the public body with which the doctor has an employment or contractual relationship. Various laws govern liability for damage caused to a public body by officials in a service relationship with it. The court before which such claims are heard is the Court of Auditors.

The Court of Cassation [58] has clarified that the employment relationship with the (now Local Health Authorities) is established not only with salaried doctors, but also with those who, through a contract, are involved in activities pertaining to the structural and procedural organisation of those very Units. Therefore, the prescription of fluoroquinolones outside the criteria of utility and necessity, as well as compromising the effectiveness of the therapeutic intervention, could also be deemed to cause damage to the public finances. There are no documented cases, but such cases cannot be ruled out in the future either. In fact, the liability asserted before the Court of Auditors concerns financial losses that may be caused to the administration not only directly (for example, a healthcare worker damaging an A.S.L. machine), but also by causing damage to a third party who then obtains compensation from the administration: consider, for instance, a case where the clinical prescription of fluoroquinolones was ill-advised and caused harm to the patient, for which the administration was subsequently required to pay compensation.

It must also be borne in mind that the clinical decision as to whether or not to follow guidelines in the patient’s best interests can be complicated by budgetary constraints, which affect the freedom to prescribe medicines. In a context characterised by limited resources and the need to contain public expenditure, healthcare professionals may find themselves in the difficult position of having to balance their decisions against budgetary constraints—decisions which should instead be guided exclusively by the principle of ‘science and conscience’ in order to realise the right to health. The most appropriate prescription for an individual case may conflict with spending targets, for which prescribing doctors may be called to account before the Court of Auditors.

### 5.3. Off-Label Use, Informed Consent and Pharmacovigilance

Whether a fluoroquinolone prescribed for a restricted indication is ‘off-label’ needs careful wording. The indication is still listed in the SmPC, but subject to a condition, namely that other commonly recommended antibacterials cannot be used. In our interpretation, when that condition is not met the prescription is not in accordance with the terms of the marketing authorisation and can be regarded as off-label; use for a purpose that the product information excludes, such as the prevention of recurrent lower UTI, is off-label in the strict sense [7]. To our knowledge the first point has not been settled by case law or by a statement of the regulator, and other readings are possible. Under Article 3 of Decree-Law no. 23/1998, converted into Law no. 94/1998, the physician must comply with the authorised indications and may depart from them only in individual cases, under his or her direct responsibility, after informing the patient and obtaining consent, when he or she considers, on the basis of documentable data, that the patient cannot be usefully treated with medicines authorised for that indication, and provided that the use is consistent with studies published in internationally accredited scientific journals [13]. The Code of Medical Ethics adds that the physician must state the reasons, obtain written informed consent and monitor the effects over time [14]. In our view, these conditions are unlikely to be met when an authorised first-line alternative is suitable for the patient.

Independently of the off-label question, Law no. 219/2017 gives every person the right to be informed in a complete, up-to-date and comprehensible way about the benefits and risks of the proposed treatment and about the possible alternatives, and requires that consent be documented in writing, or by other suitable means, and entered in the medical record [12]. For fluoroquinolones the rare but potentially disabling reactions, the early symptoms that should lead the patient to stop treatment, and the existence of alternatives are therefore material information.

Italian law also requires healthcare professionals, including physicians and pharmacists, to report suspected adverse reactions through the national pharmacovigilance network within two days of becoming aware of them, or within 36 h for biological medicines and vaccines [51]. Reporting a suspected fluoroquinolone reaction is thus a legal duty as well as good practice. A report expresses a suspicion that the medicine caused the event; it does not in itself imply fault, and it adds to the evidence on which regulatory decisions rest. In an analysis of spontaneous reports from the Campania region, risk factors, most often age over 60 years, were recorded in more than half of the reports of musculoskeletal, neurological or psychiatric reactions to quinolones [21].

### 5.4. Prescribing Appropriateness and Liability

The clinical decision as to whether or not to apply the guidelines in the patient’s best interests can be complicated by budgetary constraints, which affect the freedom to prescribe medicines. In a context characterised by limited resources and the need to contain public expenditure, healthcare professionals may find themselves in the difficult position of having to balance their decisions against budgetary constraints—decisions which should instead be guided solely by the principle of ‘science and conscience’ in order to realise the right to health. The most appropriate prescription for an individual case may conflict with expenditure targets, for which prescribers can be held to account before the Court of Auditors (administrative liability for loss to the public purse) as a result, a doctor might be inclined to prescribe treatments that are not fully suited to the patient’s condition, which could in turn expose him or her to civil or, depending on the circumstances, criminal liability. With regard to this issue, it must be reiterated that medical practice must strike a balance between the fundamental right to health (Article 32 of the Constitution) and the need to avoid the inappropriate or disproportionate use of medicines [59]. Prescribing medicines outside the criteria of utility and necessity may compromise the effectiveness of the therapeutic intervention and may also expose the healthcare professional to the risk of penalties for financial loss to the public purse, should the reimbursement of inappropriate treatments at the expense of the National Health Service be certified.

In this context, clinical guidelines represent a fundamental tool for best practice, although they do not constitute a mandatory course of action but merely a recommendation to be assessed on a case-by-case basis. However, an analysis of the scientific literature highlights significant concerns regarding the applicability of guidelines in crucial areas such as the treatment of urinary tract or respiratory tract infections, where methodological quality and attention to practical implementation issues are often lacking. Doctors should therefore not confine themselves to ‘obedience-based medicine’ or passive adherence to protocols geared solely towards cost-saving, but should exercise their technical discretion based on a meticulous assessment of the specific clinical case [60].

Finally, managing the risks associated with the inappropriate use of broad-spectrum medicines requires rigorous attention to informed consent and the assessment of the risk–benefit balance. Where a treatment is prescribed without adequate information on possible complications, or contrary to established scientific evidence and without a documented clinical justification, the conduct could, depending on the circumstances and subject to judicial assessment in the individual case, be characterised as gross negligence (*colpa grave*), generally understood as a marked departure from the rules of due care and basic technical knowledge. The degree of fault matters in Italian law: under Article 9 of Law no. 24/2017, recourse actions against the healthcare professional are admitted only in cases of wilful misconduct or gross negligence [11]. Italian case law has held that cost-containment considerations cannot prevail over the patient’s need to receive the necessary care to safeguard health and physical and mental well-being, where the right core to health as a fundamental right (guaranteed by Article 32 of the Italian Republican Constitution) is at risk [61]. In this respect, the doctor’s professional autonomy remains the fundamental safeguard to ensure that therapeutic decisions are always guided by the patient’s best interests.

## 6. Practical Strategies for Safer Prescribing in Primary Care

### 6.1. From Risk to Routine

The risks described above are inherent to fluoroquinolone exposure and cannot be eliminated once the drug has been administered. What is largely avoidable, however, is unnecessary or inappropriate exposure to these agents. These situations arise from a few recurring prescribing patterns, most notably the use of a fluoroquinolone for a mild infection when a safer first-line option is available. The same prescribing practices that reduce avoidable patient exposure also make the prescriber’s decision easier to explain and to document. The strategies below draw on outpatient antibiotic stewardship, which has been shown to reduce both overall and inappropriate fluoroquinolone use without simply shifting prescribing towards other broad-spectrum antibiotics [62].

### 6.2. Ask First Whether an Antibiotic Is Needed

The “Four Moments of Antibiotic Decision Making” framework [63] poses four plain questions:

1. “Does this patient have an infection that requires antibiotics?”
2. “What type of infection is it? Have I ordered appropriate cultures before starting antibiotics? What empirical antibiotic therapy should I initiate?”
3. “What duration of antibiotic therapy is needed for this patient’s diagnosis?”
4. “A day or more has passed. Can I stop antibiotics? Can I narrow therapy? Can I change from intravenous to oral therapy?”

This is particularly useful for the conditions most often over-treated with fluoroquinolones, many of which are self-limiting [64].

### 6.3. Reserve Fluoroquinolones and Choose a First-Line Agent

When an antibiotic is genuinely needed for uncomplicated cystitis, first-line options are nitrofurantoin, trimethoprim-sulfamethoxazole, or fosfomycin, with the fluoroquinolone held back for when these are unsuitable; the choice among first-line agents depends on local resistance patterns and availability [44,45]. This is easier when supported by a system rather than left to memory: condition-specific order sets, embedding the regulatory warning into the electronic prescription, and, where appropriate, suppressing fluoroquinolone susceptibilities on urine culture reports. In a US county health system, a multimodal programme combining these steps with education cut total outpatient fluoroquinolone prescriptions by 39% and inappropriate use from 53% to 34% [62]. Reserving is not the same as withholding. Fluoroquinolones remain appropriate, and may be the preferred oral option, for infections such as acute pyelonephritis managed in the community, where local resistance rates and susceptibility results allow, and bacterial prostatitis, and for patients in whom first-line agents are ruled out by allergy, intolerance, interactions or documented resistance [44]. Avoiding a fluoroquinolone when it is the indicated treatment can itself harm the patient and is equally inappropriate.

### 6.4. Check the Individual Patient’s Risk

Before prescribing, consider the factors that raise the risk of serious harm: age over about 60, concurrent corticosteroids, renal impairment, solid-organ transplantation, and risk factors for aortic disease [7,21,28]. Fluoroquinolones should generally be avoided in myasthenia gravis, and QT-prolonging interactions should be checked [6,25]. In a higher-risk patient the balance tips further towards a first-line alternative and that reasoning is exactly what should be recorded.

### 6.5. Inform the Patient in Plain Terms

If a fluoroquinolone is chosen, prescribing doctors should advise the patient through a brief and clear discussion: serious reactions affecting the tendons, nerves and the nervous system are rare but can be disabling and, in some cases, permanent; they may occur after a single dose or weeks to months after treatment has been stopped; furthermore, the medicine must be stopped and a doctor consulted at the first sign of pain or swelling in the tendons, new numbness or tingling, or unusual changes in mood or sleep [7,17]. Recording this discussion documents that the information was given, as Law no. 219/2017 requires [12]; when the use is off-label, written consent should be obtained (Section 5.3).

### 6.6. Support Change at the Practice Level

Individual effort lasts longer when the practice supports it. Audit and peer-comparison feedback has reduced unnecessary antibiotic prescribing in primary care in randomised trials conducted in the United States [64,65], because the drivers are mostly behavioural. Together these turn safe prescribing from an act of memory into a feature of the system.

### 6.7. The Role of Pharmacists

Pharmacists can support each of the steps above. The evidence for their contribution is growing, although most of it comes from outside Italy. In a systematic review and meta-analysis of 15 studies, stewardship interventions involving pharmacists reduced antibiotic prescribing by general practitioners (OR 0.86, 95% CI 0.78–0.95) and increased guideline-adherent prescribing (OR 1.96, 95% CI 1.56–2.45); education combined with prescribing feedback, and group meetings, were among the effective components [66]. A 20-year systematic review found audit and feedback to be the most frequently reported pharmacist-led intervention, with outpatient programmes reported mainly since 2016 [67]. For fluoroquinolones specifically, a pharmacist-led programme in a US Veterans Affairs ambulatory service combined academic detailing with a mandatory structured note that prompted the prescriber to consider alternatives and to discuss adverse effects with the patient; fluoroquinolone prescribing fell from 4.44 to 1.96 per 1000 population [68].

Prescription review and decision support are a second area. A clinical decision support tool for uncomplicated UTI, developed in a US family medicine clinic by a team that included pharmacists, was followed by a fall in fluoroquinolone use from 42% to 15% [69]. Pharmacist-led assessment of reported penicillin allergy in a US emergency department reduced fluoroquinolone use by 11% [70], which suggests that unverified beta-lactam allergy labels contribute to avoidable fluoroquinolone use. At dispensing, the pharmacist can check the prescription against the patient’s age, co-medication (corticosteroids, QT-prolonging medicines, antidiabetic agents) and history, and contact the prescriber when a risk factor or a suitable alternative is identified. Because the indication is not usually stated on the prescription, this check depends on what the patient reports and on communication with the prescriber.

Patient education and risk communication are a third area, because the community pharmacist is often the last professional the patient meets before starting treatment. In a nationwide Italian survey, 66.2% of community pharmacists reported warning clients about the potential adverse effects of antibiotics, although 37.1% reported having dispensed antibiotics without a prescription [71]; in another survey, 70.8% reported often or always informing the public about antibiotic resistance [72]. A qualitative systematic review found that time pressure and organisational constraints often limit patient education on antibiotics in primary care [73]. For fluoroquinolones, repeating at dispensing which symptoms require the patient to stop treatment and seek advice (tendon pain or swelling, numbness or tingling, changes in mood or sleep, sudden chest, abdominal or back pain) complements the information given by the prescriber. A meta-analysis of community pharmacist-led interventions found some improvement in the quality of antibiotic dispensing and counselling but concluded that the interventions, as currently implemented, are not sufficiently effective and that the studies carry a high or unclear risk of bias [74]. Pharmacists also share with physicians the duty to report suspected adverse reactions [51]; educational interventions may increase reporting by healthcare professionals, although the certainty of the evidence is low [75].

Collaboration between pharmacists and general practitioners is the model most relevant to primary care. In the meta-analysis cited above, programmes delivered by pharmacist–general practitioner teams reduced antibiotic prescribing (OR 0.93, 95% CI 0.90–0.95) and increased guideline adherence (OR 1.72, 95% CI 1.04–2.84) [66]. In Italy, where general practitioners and community pharmacies are both contracted to the National Health Service, shared local protocols for uncomplicated UTI, feedback on fluoroquinolone dispensing data and an agreed channel for querying prescriptions are feasible forms of collaboration. To our knowledge they have not been formally evaluated for fluoroquinolones in Italy, and they should be. In our view, pharmacist involvement does not transfer responsibility for the therapeutic choice, which remains with the prescriber.

### 6.8. A Proposed Checklist for Prescribing and Documentation

The steps above can be condensed into a single tool. The checklist presented in Table 3 is a proposal developed by the authors, through informal consensus and without a formal consensus method, to translate the regulatory recommendations and clinical guidance reviewed in this article into a practical framework for fluoroquinolone prescribing. Its content was synthesised from the regulatory documents, guidelines, and evidence discussed above, with particular emphasis on indication appropriateness, patient information, and documentation. The checklist has not undergone prospective validation or formal assessment of its reliability, feasibility, or clinical impact. It should therefore be regarded as a conceptual framework and a proposed, non-validated tool intended to support clinical decision-making and documentation, not as a clinical practice recommendation or a legal standard. Completing it does not guarantee protection from liability, and not completing it does not imply negligence.

Before it can be recommended for routine use, the checklist would need to be evaluated in stages. First, content validity should be established through a formal consensus process, such as a Delphi study involving general practitioners, pharmacists, infectious-disease specialists, forensic physicians and jurists, reported according to current standards for consensus methods [76]. Second, usability and inter-rater reliability should be tested by asking different clinicians to apply the checklist to the same anonymised cases. Third, feasibility should be assessed in a pilot study in general practice, including the time required and integration into the electronic health record. Finally, its effect should be measured in a controlled design, for example a cluster-randomised trial or an interrupted time-series study, on outcomes such as the proportion of fluoroquinolone prescriptions that meet the product-information conditions, the completeness of the documentation of indication and consent, and the reporting of suspected adverse reactions. Any effect on litigation would require long-term observation and is unlikely to be measurable in the short term.

**Figure 2:**
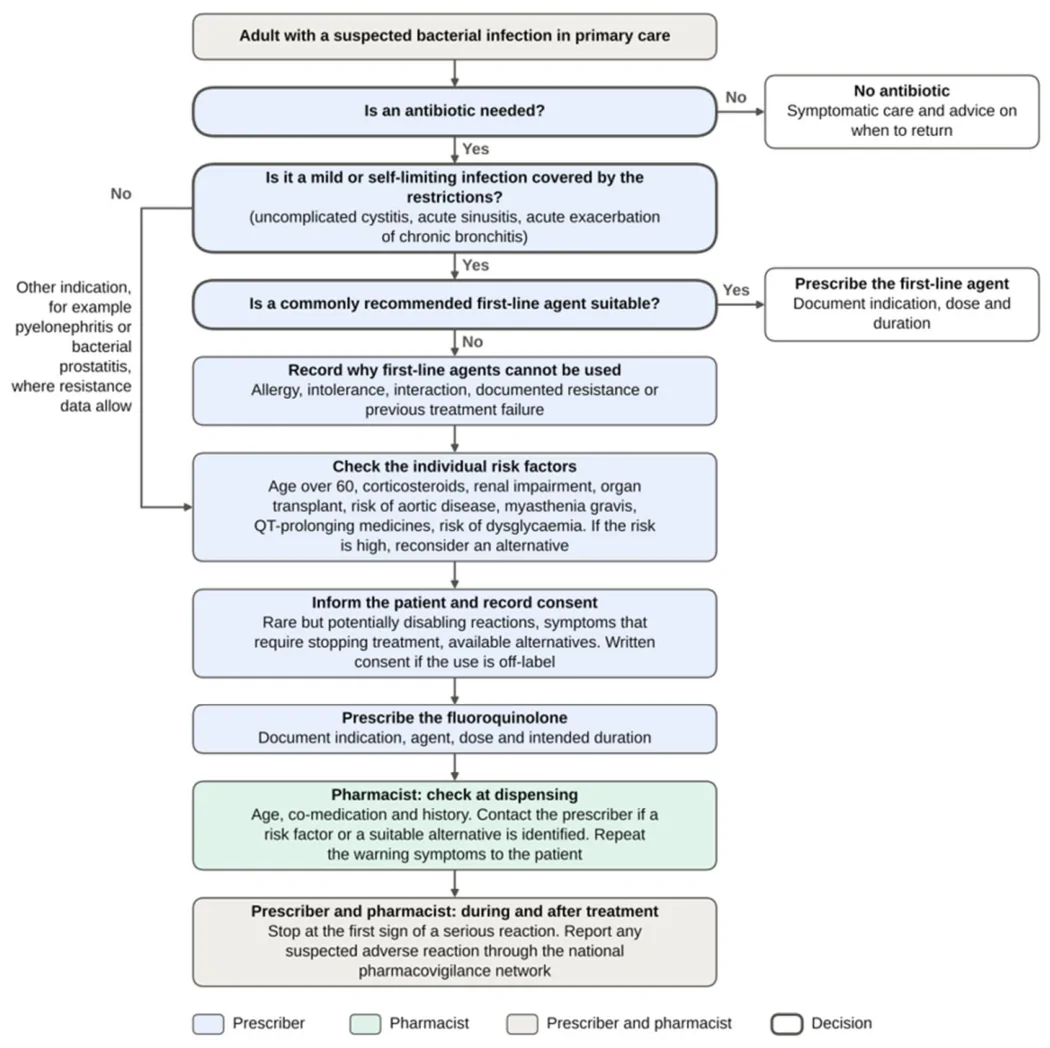
Proposed pathway for fluoroquinolone prescribing in primary care, including the pharmacist’s check at dispensing. The pathway summarises Section 6.2, Section 6.3, Section 6.4, Section 6.5, Section 6.6, Section 6.7 and Section 6.8 and Table 3. It reflects the authors’ informal consensus, has not been validated, and is not a clinical practice recommendation. “Restrictions” refers to the conditions of use in the European product information [7].

The same steps are shown as a pathway in Figure 2, which also places the pharmacist’s check at dispensing.

## 7. Discussion

### 7.1. Main Findings

This review brings together three strands usually discussed separately: the safety profile of fluoroquinolones, the way they are prescribed in general practice, and the forensic consequences. Regulators on both sides of the Atlantic have concluded that, for uncomplicated UTI, acute sinusitis, and acute exacerbation of chronic bronchitis, the risks generally outweigh the benefits and the drugs should be reserved for patients with no suitable alternative. Yet, in the countries for which data are available, much primary-care prescribing continues for exactly these conditions: uncomplicated cystitis accounted for most inappropriate use in the US studies [8,62], and in the Italian REWIND data ciprofloxacin was given in about a quarter of antibiotic-treated UTIs [47]. It is this persistent gap that gives the topic its forensic relevance.

### 7.2. Putting the Safety Signal in Perspective

A key message is one of proportion. The adverse events underlying these warnings are serious, but their absolute frequency is low, and, as Table 1 shows, the evidence behind them is not equally strong for every reaction. A systematic review and meta-analysis found an increased risk of aortic aneurysm or dissection with current fluoroquinolone use, while also showing that the absolute excess risk was considerably smaller: assuming a baseline incidence of 10 events per 100,000 patient-years, 7246 patients would need to be treated for at least three days for one additional event to occur [36]. This estimate is population-dependent, however, and should not be interpreted as a fixed risk for individual patients. Moreover, the observational evidence is susceptible to residual confounding and surveillance bias. In a large propensity score-matched cohort, the association varied according to the clinical indication, being observed in patients treated for pneumonia but not in those treated for UTI; absolute event rates remained below 0.1% in both cohorts [30]. The association was also substantially attenuated when baseline aortic imaging was taken into account, supporting the possibility that differential surveillance contributes to the observed signal. This nuance matters in both directions. Fluoroquinolones remain valuable and appropriate for selected serious infections, where their potential benefits may outweigh a small potential increase in risk. From a forensic perspective, the occurrence of an adverse event alone does not establish negligence. Professional conduct must instead be assessed considering the indication, available alternatives, information provided to the patient, and the reasonableness of the clinical decision at the time of prescribing. The reasonable position is therefore not to avoid fluoroquinolones altogether, but to use them when clinically justified and to document the clinical reasoning underlying their use.

### 7.3. Why the Gap Persists

The gap is behavioural more than informational. Warnings alone have had only a modest, uneven effect on outpatient prescribing [8,9,10]; the largest reductions have followed active stewardship rather than warnings in isolation [62,68]. Some prescribers still believe fluoroquinolones outperform first-line agents for cystitis, a belief the guidelines do not support [48]. This helps explain why system-level changes—order sets, default alerts, suppressed susceptibility reporting, and peer feedback—tend to outperform education alone [62,64]. Not every such measure works equally well: in a US cluster-randomised trial, accountable justification and peer comparison reduced inappropriate antibiotic prescribing, whereas order sets suggesting alternatives did not reach statistical significance [65]. Most of this evidence comes from the United States. Whether it applies to Italian general practice, which differs in organisation, prescribing software and resistance patterns, cannot be assumed and needs local evaluation. AI-based decision support may in future help with documentation and clinical decisions, but it raises ethical questions of its own and has not been evaluated for fluoroquinolone prescribing [77].

### 7.4. Implications for General Practice and Community Pharmacy

The practical implications are modest and achievable. Treat uncomplicated cystitis, sinusitis, and bronchitis as conditions where a fluoroquinolone should rarely be first choice and reserve the class for cases where first-line options are genuinely unsuitable. When one is used, check the individual risk factors, inform the patient in plain language, and record all of it. These steps are simultaneously good clinical care and a sound basis for accounting for the decision, because they align the prescription with the product information, the guidelines and the duty to inform; they do not guarantee protection from liability. For pharmacists, the implications are to review fluoroquinolone prescriptions for risk factors, to reinforce the safety information at dispensing, to report suspected reactions, and to take part in local stewardship with general practitioners. Table 3 is intended to make these steps easy to apply.

### 7.5. Limitations

This is a narrative rather than a systematic review, with no formal quality appraisal or quantitative synthesis. The search was broad, the records were not screened exhaustively, and the choice of sources reflects the authors’ judgement, so relevant studies may have been missed and selection bias cannot be excluded. Much of the prescribing and safety evidence comes from the United States and northern Europe and may not transfer to every health system; Italian data at the level of the indication are limited. The forensic analysis is confined to the Italian framework, and rules differ between jurisdictions; it reflects the authors’ reading of statutes and case law, some points remain open to interpretation, and it is not legal advice. The evidence on the role of pharmacists comes largely from non-Italian settings and is of variable quality. The proposed checklist has not been validated. Finally, the evidence base is still evolving, particularly for the neuropsychiatric and cardiovascular signals, so risk estimates should be treated as provisional.

## 8. Conclusions

Fluoroquinolones are effective antibiotics whose rare but serious, potentially irreversible adverse effects have led regulators to restrict their use in common, self-limiting infections. In general practice a gap persists between these recommendations and everyday prescribing, driven largely by cystitis—and this gap is where avoidable patient harm and potential prescriber liability meet. The way to close it is neither to abandon a useful drug class nor to fear it, but to prescribe it deliberately: for the right indication, in the right patient, after informing them, and with the reasoning recorded. Stewardship and appropriate use are two sides of the same principle: restricting fluoroquinolones where safer alternatives exist is what preserves them, in terms of both safety and resistance, for the patients in whom they are clinically indicated, and those patients should not be denied them. Doing so protects the patient and, under the Italian framework examined here, allows the physician to account for the decision. Pharmacists can reinforce each of these steps. The proposed checklist in Table 3 offers a framework for applying this principle; it has not been validated and should be formally evaluated before routine use.

## Figures and Tables

**Table 2:** Sources relevant to fluoroquinolone prescribing and their status under the Italian legal framework.

Type of Source	Examples Relevant to Fluoroquinolones	Status and Role in Assessing Liability
Legal norms	Law no. 24/2017 (safety of care and professional liability); Law no. 219/2017 (informed consent); Decree-Law no. 23/1998, converted into Law no. 94/1998, Art. 3 (off-label use); Ministerial Decree of 30 April 2015 (pharmacovigilance) [11,12,13,51]	Binding. They set the professional’s duties (to follow accredited guidelines save for the specific features of the case, to inform and obtain consent, to respect the authorised indications or the conditions for off-label use, to report suspected adverse reactions) and the criteria of liability
Regulatory provisions	European Commission decision following the EMA review (2019) and the resulting SmPC; AIFA *Note Informative Importanti*. For comparison only: FDA boxed warning [6,7,37]	The SmPC is part of the marketing authorisation and defines the authorised conditions of use; a prescription outside those conditions may fall under the off-label regime (Section 5.3). Safety communications inform prescribers of the risk and of changes to the product information and are relevant to the foreseeability of harm. FDA labelling has no legal effect in Italy and counts only as scientific evidence
Clinical guidelines and good clinical practice	Guidelines published in the National Guideline System (SNLG) under Art. 5 of Law no. 24/2017; international guidelines such as those of the European Association of Urology [11,44,45]	Not binding. SNLG guidelines are the parameter referred to in Arts. 5 and 6 of Law no. 24/2017; in their absence, good clinical-care practices apply, to which international guidelines contribute. The professional must depart from them when they do not fit the individual case [52,53]
Forensic interpretation	Case law, forensic doctrine, expert opinion in the individual case; the analysis and the checklist proposed in this review [52,53,54]	No normative force. It applies the sources above to the facts of a specific case (fault, harm and causal link). Its conclusions are case-specific and cannot be generalised

EMA, European Medicines Agency; SmPC, summary of product characteristics; AIFA, Italian Medicines Agency; FDA, US Food and Drug Administration; SNLG, *Sistema Nazionale Linee Guida*. The right-hand column reflects the authors’ reading of the sources and is not legal advice.

**Table 3:** Proposed, non-validated checklist for fluoroquinolone prescribing in primary care (authors’ informal consensus; not a clinical practice recommendation).

Checklist Item (Action)	Clinical/Regulatory Rationale	Forensic Function
Confirm a valid indication, concordant with the product information and guidelines; avoid fluoroquinolones for uncomplicated cystitis, acute sinusitis, and acute exacerbation of chronic bronchitis when other options exist	A substantial share of outpatient fluoroquinolone use is for conditions for which it is not recommended, often self-limiting [43,62]	Documents the appropriateness of the indication
Verify that safer first-line alternatives were considered and documented as unsuitable	The European product information and the FDA labelling restrict fluoroquinolones to cases where alternatives cannot be used [6,7]	Shows that the conditions of authorised use were considered
Screen for and record key risk factors: age > 60, renal impairment, organ transplantation, myasthenia gravis, aortic aneurysm risk, concomitant corticosteroids, prior tendinopathy, QT-prolonging drugs, dysglycaemia risk	Associated with aortic aneurysm/dissection, tendinopathy/rupture, QT prolongation, and dysglycaemia, often early (Table 1)	Shows that foreseeable risks were assessed
Inform the patient and record informed consent, covering disabling and potentially irreversible effects and the available alternatives; obtain written consent for any off-label use	Law no. 219/2017 requires information on benefits, risks and alternatives; off-label use requires consent under Law no. 94/1998 [12,13]	Documents that the duty to inform was addressed
Document the specific indication, agent, dose, and intended duration	A documented indication makes the decision open to later review	Provides a contemporaneous record of the decision and its reasons
Provide safety-netting: advise stopping the drug and seeking care for tendon pain, new neuropathy, chest/abdominal/back pain, palpitations, or neuropsychiatric symptoms; document it	Regulators advise stopping treatment at the first sign of a serious reaction [7]	Documents follow-up advice and continuity of care
Report any suspected adverse reaction through the national pharmacovigilance network	Reporting is mandatory for healthcare professionals and informs regulatory decisions [51]	Fulfils a legal duty (Ministerial Decree of 30 April 2015)

The checklist was developed by informal consensus among the authors from the sources reviewed in this article. It has not undergone prospective validation or any assessment of reliability, feasibility or clinical impact. The ‘Forensic function’ column reflects the authors’ interpretation under the Italian legal framework; completing the checklist does not exclude liability. The risk-factor check, the safety advice and the reporting of suspected reactions can also be supported by the pharmacist at dispensing (Section 6.7).

## Data Availability

No new data were created or analysed in this study. Data sharing is not applicable to this article.
